# Impact of CT dose on AI performance: A comparison of radiomics, deep, and foundation models in a multicentric anthropomorphic phantom study

**DOI:** 10.1002/mp.70374

**Published:** 2026-03-18

**Authors:** María Martín Asiain, Mohammadreza Amirian, Oscar Jimenez del Toro, Christoph Aberle, Roger Schaer, Michael Bach, Markus Obmann, Kyriakos Flouris, Henning Müller, Bram Stieltjes, Ender Konukoglu, Vincent Andrearczyk, Adrien Depeursinge

**Affiliations:** ^1^ Institute of Informatics, School of Management HES‐SO Valais‐Wallis University of Applied Sciences and Arts Western Switzerland Sierre Switzerland; ^2^ Nuclear Medicine and Molecular Imaging Department Lausanne University Hospital Lausanne Switzerland; ^3^ Idiap Research Institute Martigny Switzerland; ^4^ Clinic of Radiology and Nuclear Medicine, University Hospital Basel University of Basel Basel Switzerland; ^5^ Computer Vision Lab ETH Zurich Zurich Switzerland; ^6^ Faculty of Medicine University of Geneva (UNIGE) Geneva Switzerland

**Keywords:** computed tomography, radiation dose, radiomics

## Abstract

**Background:**

Computed tomography (CT) is widely used in clinical practice due to its ability to provide detailed anatomical information. However, variations in radiation dose can affect image quality, potentially compromising the performance and reliability of artificial intelligence (AI) models applied to these images.

**Purpose:**

To evaluate the robustness of radiomics‐based and deep learning‐based models to variations in CT dose levels using a standardized dataset obtained from a 3D‐printed anthropomorphic phantom simulating liver tissue with anomalies, as well as in the publicly available dataset CT‐ORG with real patient data for organ classification. This study is in an early experimental stage, tested only on retrospective data.

**Methods:**

A total of 1378 image series from 649 scans were acquired across 13 scanners from four manufacturers at five dose levels. Features were extracted from six regions of interest (ROIs), representing four liver tissue types (normal, cyst, hemangioma, metastasis), using four methods: PyRadiomics, a shallow convolutional neural network (CNN), SwinUNETR, and a CT foundation model (CT‐FM). Feature stability was assessed using the Intraclass Correlation Coefficient (ICC), while Uniform Manifold Approximation and Projection (UMAP) was employed to evaluate tissue types separability and the influence of scanner variations. Generalizability was tested by training liver tissue classifiers on one dose level and testing on others, alongside a dose classification task (10‐fold cross‐validation) to determine the sensitivity of each method to dose variations. In addition, we compared the four methods in addressing the task of organ classification (10‐fold cross‐validation) with the CT‐ORG dataset containing 140 CT scans acquired with varying dose levels.

**Results:**

Radiomic features showed limited robustness to dose variations, leading to reduced performance in liver tissue classification and the lowest ICC among methods (ICC: 0.8355 ± 0.1705). SwinUNETR and CT‐FM exhibited the highest stability (SwinUNETR ICC: 0.9528 ± 0.0272; CT‐FM ICC: 0.9347 ± 0.0420), clearly above the Shallow CNN (ICC: 0.8416 ± 0.2018). CT‐FM also showed strong generalization across dose levels: its features effectively distinguished between liver tissue types and dose levels simultaneously, without compromising performance in either task. Consistent with these trends in dose sensitivity, CT‐FM obtained the highest dose‐classification accuracy (0.6517 ± 0.0179), whereas SwinUNETR showed the lowest (0.3796 ± 0.0250). These trends were confirmed in the context of organ classification with real patient data on the CT‐ORG dataset, where CT‐FM achieved the highest accuracy (0.965).

**Conclusions:**

The study highlights the limited robustness of traditional radiomics and deep models to CT dose variation and underscores the potential of foundation models like CT‐FM to enable robust clinical applications by mitigating dose‐related variability. This enhanced performance is likely due to the model's pretraining on large and diverse datasets, allowing it to learn robust and generalizable representations across varying acquisition conditions.

## 1 INTRODUCTION

Computed tomography (CT) is a widely used medical imaging technique that enables detailed visualization of internal structures. However, the radiation dose used during image acquisition can significantly impact image quality, affecting noise levels, contrast, and overall diagnostic performance. In clinical practice, dose levels are adapted based on the diagnostic task, the characteristics of the patient, and the equipment settings. While lower doses are preferred to reduce radiation exposure, they often result in noisier images and reduced resolution, which can hinder clinical interpretation.

The impact of CT dose on image quality can potentially influence the performance of machine learning (ML) models used in medical image analysis. Previous studies suggested that handcrafted features may be particularly sensitive to these variations,^1^ while certain deep learning (DL) models show signs of greater resilience.^2^ However, comprehensive comparisons across different model types and complexities remain limited, and further investigation is needed to understand how dose variations impact various feature extraction methods and predictive models.

A key challenge in this context is isolating the sources of variability—such as anatomical, physiological, and technical factors—that may affect feature stability and model performance. An important advantage of using phantoms is their ability to eliminate inter‐ and intra‐patient variability, allowing us to isolate the specific impact of dose. Unlike simple quality control phantoms, anthropomorphic phantoms closely mimic human tissue and organ structures, enabling more realistic and clinically relevant evaluations.

In this study, we investigate the impact of CT dose variation on feature extraction and predictive performance across multiple model types. We evaluate four feature extraction strategies: 1st and 2nd order radiomics (PyRadiomics); a shallow convolutional neural network (CNN)^3^; the encoder of the SwinUNETR model, as described by Tang et al.^4^; and a CT Foundation Model (CT‐FM), a large‐scale model introduced by Pai et al.^5^ Our analysis is based on phantom scans acquired across five different dose levels at multiple centers using various CT scanners, with features extracted from six regions of interest (ROIs). We also evaluate whether CT‐FM, as a foundation model, offers improved built‐in robustness to dose‐related degradation compared to conventional radiomics and other DL methods.

## 2 RELATED WORK

Robustness to data acquisition variability is a key concern in medical image analysis, especially when using quantitative features for downstream predictive tasks. CT imaging, in particular, is sensitive to changes in dose, reconstruction parameters, and scanner hardware, which can lead to shifts in image distributions and reduce the generalizability of models across sources. Understanding how dose reduction and acquisition parameters affect both handcrafted and learned features is critical, especially as clinical efforts increasingly aim to reduce patient radiation exposure.^6^

Meyer et al.^1^ investigated the effect of varying dose and reconstruction settings on radiomic feature reproducibility using CT scans from a prospective clinical trial. Rather than acquiring multiple exposures, the study exploited the dual‐source architecture of a second‐generation CT scanner, in which the two X‐ray tubes were operated at the same voltage but different currents. By linearly combining the projection data from tube A and tube B with varying weighting factors, the authors generated images corresponding to seven distinct dose levels (ranging from 25% to 100% of the clinical dose) without additional patient exposure. This strategy ensured consistent anatomy while allowing controlled noise variation. Their analysis showed that most radiomic features were highly sensitive to acquisition parameters, with only 11% remaining consistent across all settings. Slice thickness was identified as the most influential factor. However, the study used only one scanner at a single center and did not include repeated measurements, limiting the generalizability of their findings. Moreover, the analysis focused solely on feature reproducibility and did not evaluate the implications of these variations on classification or prediction performance.

The impact of radiation dose on predictive performance has also been explored for DL models. Peters et al.^2^ examined how dose reduction affected a CNN trained to estimate malignancy risk in pulmonary nodules. Their study used simulated low‐dose CT scans, where statistical noise was added to the images to create reduced dose levels, including 25% and 5% of the original dose. The study quantified performance decline by analyzing changes in the Lung Cancer Prediction (LCP) score and risk group classification: for example, the proportion of nodules classified as high‐risk decreased from 58% at full dose to 52% at 5% dose, and a small fraction of nodules were misclassified into medium‐ or low‐risk groups. These results illustrate that CNN performance can degrade under dose‐reduced conditions, highlighting sensitivity to image quality. Notably, the study evaluated only a single model and did not examine how model complexity (i.e., number of parameters) and pretraining might influence robustness to such variations.

Other comparisons have focused on feature reproducibility across methodologies. Ziegelmayer et al.^7^ evaluated feature robustness under realistic acquisition variability by scanning nonhuman phantoms on three different CT scanners using varying protocols, including explicit modulation of tube voltage (90/120 kV and 100/120 kV), which altered tube current and radiation dose. Their comparison of handcrafted radiomic features with a pretrained VGG19 CNN revealed that CNN features demonstrated significantly greater stability, as radiomic features were highly sensitive to acquisition parameters such as tube voltage and current. This study was complemented by an in vivo analysis using CT scans from patients with hepatocellular carcinoma and with hepatic metastases from colon cancer, showing that CNN features outperformed radiomics in both reproducibility and tumor differentiation. The findings suggest that CNN‐based features are less susceptible to minor intensity fluctuations, offering higher sensitivity, specificity, and robustness, making them a promising alternative to traditional radiomics in clinical and multicenter settings, especially where scan protocols vary.

Similarly, Dehbozorgi et al.^8^ compared statistical features (e.g., mean, standard deviation, local binary pattern, Gray Level Co‐occurrence Matrix, and Histogram of Oriented Gradients), radiomic features extracted with PyRadiomics, and DL features from CNNs. They used these extracted features as input for PCA‐LDA models, which served as the binary classifiers for the classification tasks. The model performance was measured via mean sensitivity and across multiple medical imaging datasets, including H&E‐stained tissue images of colorectal cancer, chest X‐ray images, and optical coherence tomography scans. The results showed that DL models, particularly DenseNet121 and ResNet50, outperformed radiomics in terms of both accuracy and robustness to image quality variations.

In contrast to previous studies, our work specifically explores the effect of CT dose reduction on radiomic and DL features, including foundation models, with a focus on model robustness across different levels of complexity and pretraining. Rather than relying on simulated dose reduction, we analyze real‐world dose variations with the use of an anthropomorphic phantom. Using a large, multicentric dataset, we isolate the impact of acquisition changes by examining the stability of various feature types—from handcrafted radiomics to features from small neural networks and foundation models—and assess how these variations affect both feature robustness and downstream model performance.

## 3 METHODS

### 3.1 Datasets

#### 3.1.1 CT4Harmonization

**FIGURE 1:**
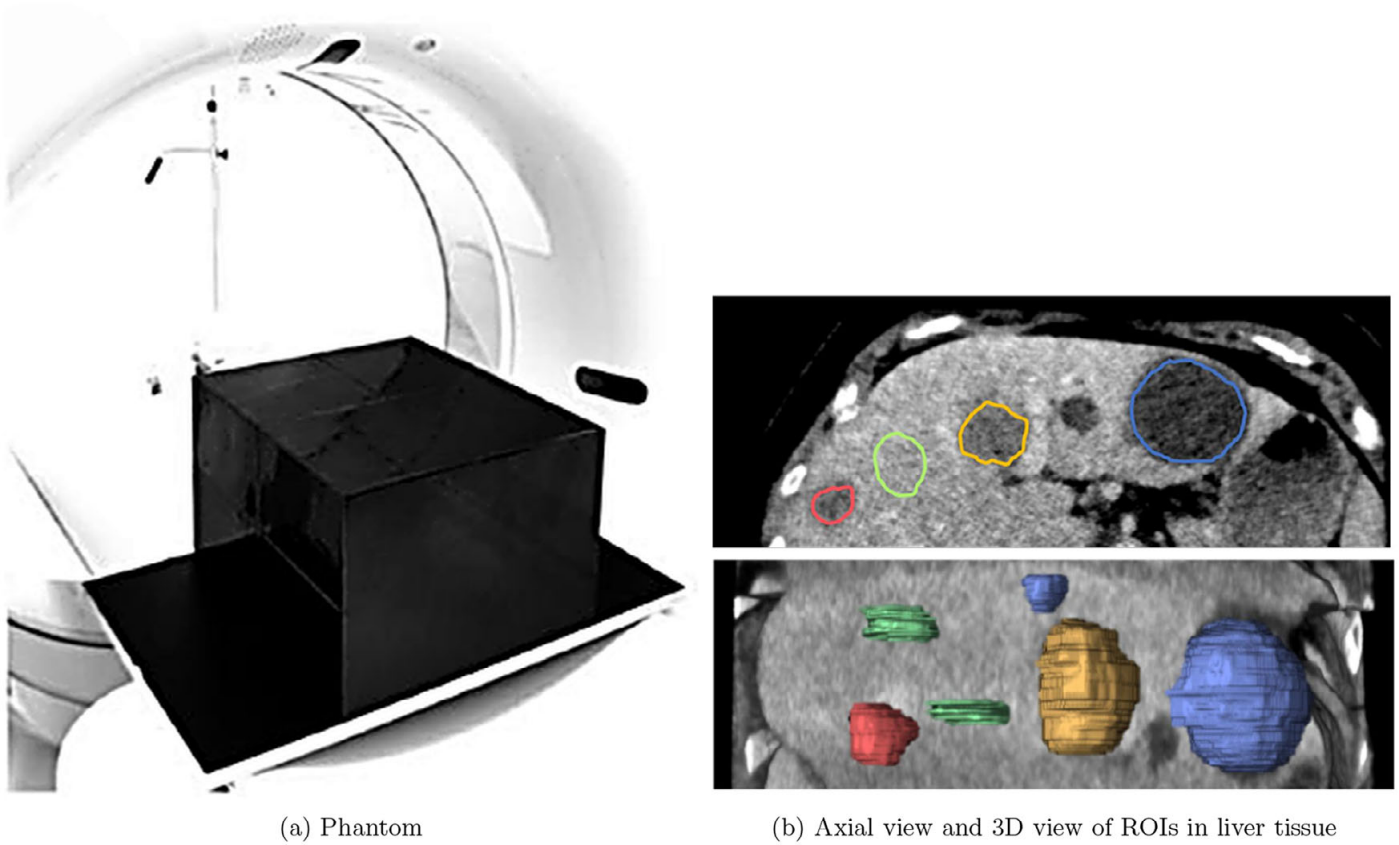
Visual overview of the anthropomorphic CT phantom used in this study. (a) Phantom and scanner.^10^ (b) Example of segmentation (six ROIs) of liver tissue proposed by human experts with four classes: cyst (blue), hemangioma (yellow), metastasis from a colon carcinoma (red) and normal (green), from Jimenez‐del‐Toro et al.^11^

The dataset was generated using a 3D‐printed phantom^9^, ^10^ infused with iodine ink, simulating the X‐ray attenuation properties of human liver tissue (Figure 1). The phantom includes four distinct anomaly regions corresponding to three different tissue types: 1 hemangioma, 2 cysts, and 1 metastasis, as well as 2 normal ROIs representing healthy liver tissue. All ROIs were manually delineated by a board‐certified radiologist (M.M.O.). The phantom was designed based on real patient data, as shown in Figure 1.

The dataset consists of 1378 CT image series from 649 CT scans of the same phantom, acquired using 13 different scanners from four manufacturers (Siemens, Philips, Toshiba and GE Medical Systems) across eight institutions (see Table S1 for detailed scanner models and configurations). The scans were acquired using a harmonized protocol and repeated at five dose levels (CTDI_vol_ = 1, 3, 6, 10, and 14 mGy). The dataset can be found on The Cancer Imaging Archive (TCIA) under the name CT4Harmonization‐Multicentric.^12^

This protocol was established following a survey on typical acquisition and reconstruction parameters used in clinical thoracoabdominal CT exams (tube voltage 120 kV, pitch 1.0, rotation time 0.5 s, collimation 40mm, field of view 350mm). The survey included 21 CT scanners from 9 centers across Switzerland, from which a harmonized set of acquisition and reconstruction parameters representing realistic clinical settings was derived, including the investigated dose levels (CTDI_vol_ = 1–14 mGy).^9^ Our maximum CTDI_vol_ of 14 mGy is intentionally kept below the ACR reference level of 25 mGy for adult abdominal CT,^13^ supporting the clinical plausibility of the selected dose range. Due to vendor‐specific constraints, it was not possible to apply identical settings across all CT scanners, resulting in slight deviations from the harmonized protocol. The tube current time product was adjusted to set the various dose levels, all other parameters were kept as similar as possible to the harmonized protocol.

**FIGURE 2:**
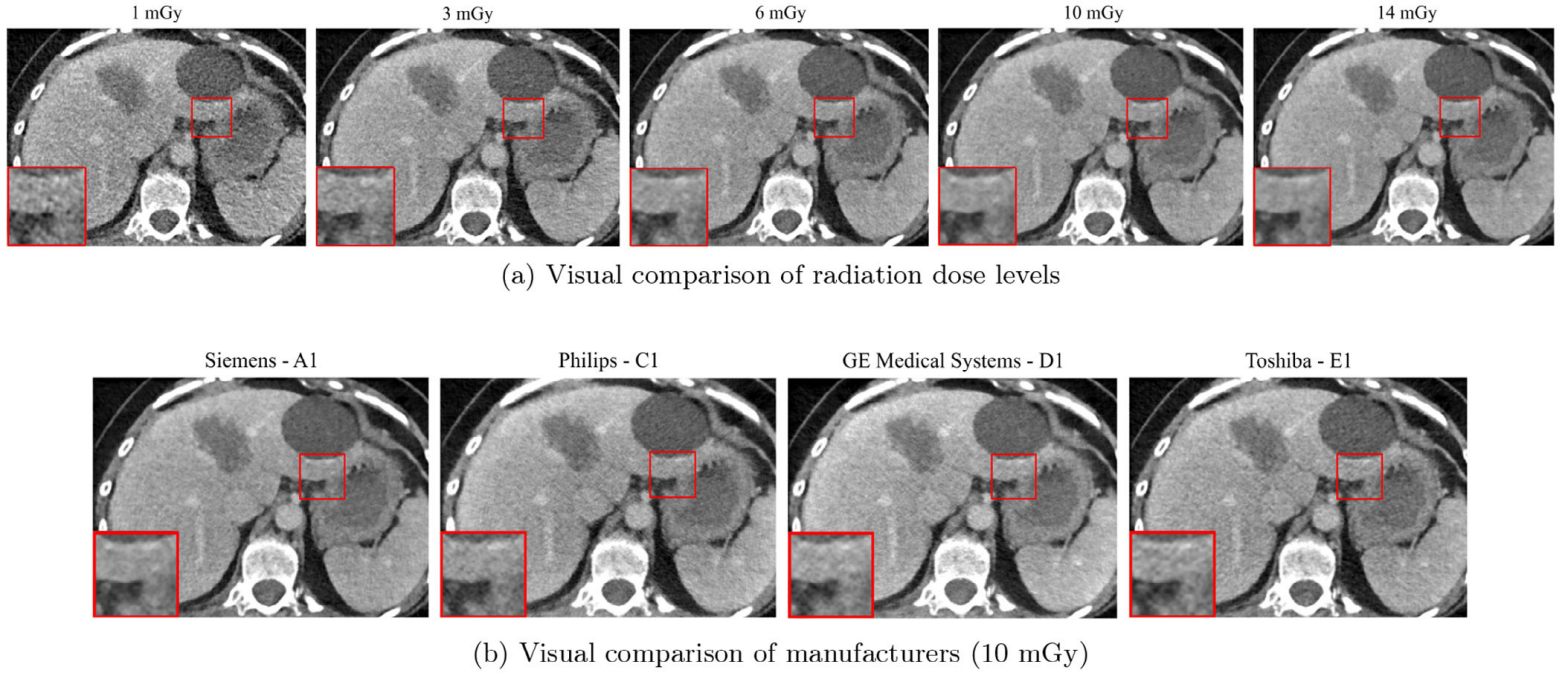
Axial CT slices acquired under two experimental settings. (a) Same scanner (Siemens SOMATOM definition Edge ‐ A1) with IR reconstruction at different radiation dose levels (1, 3, 6, 10, and 14 mGy). (b) Same radiation dose (10 mGy) and IR reconstruction, but across four different manufacturers (Siemens, Philips, GE Medical Systems, and Toshiba). A zoomed‐in patch is shown in each image to highlight texture differences for qualitative comparison (level=50, window=400).

An example of the visual impact of dose level and scanner choice on image texture and noise is shown in Figure 2: panel (a) displays axial slices from the same scanner at varying dose levels, whereas panel (b) shows slices acquired at 10 mGy on different CT scanners.

For each CT scanner and dose level, 10 repeated scans with identical settings were performed, except for the Toshiba Aquilion Prime SP scanner at 10 mGy, which only had 9 repeated scans. The resulting image series were reconstructed using two to three different algorithms. Specifically, each CT scan was reconstructed using vendor‐specific iterative reconstruction (IR) and filtered back‐projection (FBP) with a standard soft tissue kernel, yielding 649 IR and 649 FBP series. Additionally, for two scanners, a DL‐based reconstruction algorithm was available. To mitigate variations attributed to voxel dimensions, scans whose voxel geometry differed from 2 mm slice thickness and 0.684 mm pixel spacing were resampled to these values, which corresponded to the voxel size used by most scanners.

For all analyses and metric evaluations, we focused on six ROIs from four liver tissue classes covering the full range of tissue types represented in the phantom. The two cysts ROIs displayed some variability, mostly in terms of size. With a fixed patch size, the smaller cyst includes a larger fraction of boundary voxels, which can influence the extracted features. The two normal tissue regions were visually highly consistent, supporting their consolidation into one class. This grouping allowed for a more streamlined and balanced assessment across tissue types.

#### 3.1.2 CT‐ORG

To assess the generalizability of the models beyond controlled phantom experiments, we evaluated them on the CT‐ORG dataset,^14^ which includes 140 CT scans with organ segmentations across various imaging conditions. The dataset contains both contrast‐enhanced and non‐contrast scans from abdominal, full‐body, and PET‐CT exams, though exact dose values are unavailable. The available segmentations include the liver, bladder, lungs, kidneys, bones, and, in some cases, the brain. We extracted 3D patches of size (64, 64, 32) centered at the organ masks' center of mass, generating separate patches for left and right lungs and kidneys. Features were then computed using all four methods: PyRadiomics, Shallow CNN, SwinUNETR, and CT‐FM. For PyRadiomics, we used binary masks matching the patch size to ensure consistent input across methods.

### 3.2 Feature extraction methods

Features were extracted from the six ROIs corresponding to the four tissue types in each scan. We employed four feature extraction methods: PyRadiomics, a shallow CNN, the encoder of SwinUNETR, and CT‐FM.

**PyRadiomic features**: Standard radiomic features were extracted using the PyRadiomics library.^15^ These features include first‐order (intensity) and second‐order (texture) statistics of the ROIs. Shape features were excluded, as the ROI contours are fixed and not affected by dose variations. Overall, 86 features were obtained.

**CNN‐based features**: A shallow CNN model was used to extract features from the images.^3^ This model consists of two convolutional layers, two fully connected layers, and max‐pooling between the convolutional layers. It was trained on classification tasks involving anatomical structures similar to those in the phantom dataset, using CT scans. The model was pretrained on a public dataset from the VISCERAL challenge,^16^ which includes 60 CT scans annotated for anatomical structure detection and segmentation. For our feature extraction, only an early part of the network was used—specifically up to the first batch normalization layer following the second convolution—excluding the fully connected and later normalization layers. As a result, 260k parameters were involved, and the extracted representation consisted of 2048 features.

**SwinUNETR‐based features**: We used SwinUNETR,^4^ a transformer‐based model for extracting DL features. It integrates a Swin Transformer encoder with a CNN decoder for 3D medical image segmentation. The encoder processes inputs as 3D tokens through Swin Transformer blocks. The model was pretrained on 5050 publicly available CT scans using self‐supervised learning and fine‐tuned for 30 epochs in the original study to ensure generalization across scanners. While the full SwinUNETR model has 62M parameters, the encoder comprises only 8M parameters and generates 768‐dimensional feature vectors.

**CT‐FM features**: We extracted features from the CT‐FM,^5^ a large‐scale pretrained foundation model designed for diverse radiological tasks. CT‐FM was pretrained on 148,000 CT scans using a contrastive learning approach and has demonstrated strong generalization across multiple radiological applications such as tumor segmentation, head CT triage, and semantic understanding. In this study, we extracted features from the embeddings produced by CT‐FM to evaluate its robustness to dose variations. The CT‐FM framework integrates a SegResNet encoder with contrastive pretraining, with the goal of learning embeddings that are more interpretable and better suited for downstream segmentation tasks. The CT‐FM model has 77M parameters and produces 512 features.

It is worth noting that all three DL models were trained on external CT datasets of various size and sources, allowing us to focus on generalization capabilities of their representations. The four feature extraction approaches differ in their architectures, pretraining strategies, feature dimensionality, and feature extraction approaches. Specifically, PyRadiomics computes features directly from the segmentation masks of each ROI, whereas the shallow CNN, SwinUNETR, and CT‐FM extract features from 3D patches of size 64×64×32 centered on the ROIs. Importantly, the feature dimensionality is fixed by each extractor and was not tuned: PyRadiomics yields 86 features; the shallow CNN, 2048; SwinUNETR, 768; and CT‐FM, 512. These dimensions are determined by the network architecture and the specific layer from which features are extracted. A summary of the feature extraction methods and the models used is provided in Table 1.

**TABLE 1:** Summary of the feature extraction methods, number of parameters, feature dimensionality, and size of the pretraining dataset used.

Features	# Parameters	Feature size	Pretraining data size
PyRadiomics	N/A	86	N/A
Shallow CNN	260,032	2048	60 CT
SwinUNETR	8,062,002	768	5050 CT
CT‐FM	77,760,992	512	148,000 CT

### 3.3 Feature stability assessment

To evaluate the impact of dose variation on feature stability, we computed the Intraclass Correlation Coefficient (ICC) using the ICC(3,k) model^17^ for each feature independently. In our setup, dose levels act as fixed raters. We consider every combination of scanner, ROI, reconstruction method, and repetition. This gives 1,680 candidate targets across devices and settings. After keeping only those targets that are present at all five doses, we retain 1608 targets. This model assumes a fixed set of raters and is appropriate when the same dose settings are applied consistently across targets, allowing us to assess the reliability of the average rating across all dose levels. This analysis allows us to quantify how consistently features are measured across varying dose conditions. A high ICC value (close to 1) indicates that a feature remains stable across different dose levels, while lower ICC values suggest that dose variations introduce inconsistency.

The ICC is computed using the following formula:

(1) $$ICC\left(3,k\right)=\frac{MSB-MSE}{MSB},$$

where,

- MSB represents the Mean Square Between dose levels, which quantifies how much variance exists between the different doses.
- MSE represents the Mean Square Error within dose levels, which quantifies the variance within each dose group.

### 3.4 Feature separability assessment

We used data visualization methods to analyze feature stability and separability, evaluating the impact of various parameters across different models for liver tissue and scanner classification. To this end, we employed Uniform Manifold Approximation and Projection (UMAP) plots, which reduce the high‐dimensional feature space to two dimensions. Each point on the plot corresponds to a projected feature vector from the ROIs. For these visualizations, UMAP was applied using 20 neighbors, a minimum distance of 0.5 and a seed of 24. The resulting plots provide insights into how each parameter—liver tissue class, scanner manufacturer, and dose—affects the feature distribution in the feature space.

### 3.5 Liver tissue classification across dose levels

To evaluate the generalization ability of liver tissue classification models across dose levels, we conducted experiments where classifiers trained on one dose were evaluated on the remaining doses. This approach allows us to analyze how well the information obtained at a specific dose generalizes to other dose levels. The classification task involves four different liver tissue types: normal tissue, cyst, metastasis, and hemangioma, using five dose levels (1, 3, 6, 10, and 14 mGy).

For this task, we employed a MultiLayer Perceptron (MLP) classifier with four layers and a dropout rate of 0.2. The model was trained for 30 epochs with a batch size of 8, using the AdamW optimizer with a learning rate and weight decay of 1e‐4, and a categorical cross‐entropy loss.

### 3.6 Dose classification

To assess how sensitive each model's features are to radiation dose variations, we defined a dose classification task in which the goal is to predict the acquisition dose level from the extracted features. This task evaluates the extent to which the learned feature representations encode information related to the dose, rather than tissue‐specific properties as investigated in the liver tissue classification task. The five dose levels (1, 3, 6, 10, and 14 mGy) constitute the targeted classes.

The dose classification was performed using a similar MLP classifier. The data were divided into 10 folds for cross‐validation (CV) while ensuring that all samples from a given ROI acquired by a specific scanner—including its various repetitions, reconstructions, and dose levels—were grouped in the same fold. As an example, for scanner A1 and the hemangioma ROI, all features from 5 dose levels, 10 repeated scans per dose, and 2–3 reconstruction methods (e.g., IR and FBP) were included together in the same fold, yielding approximately 100 samples. This division ensures that samples from the same anatomical region and scanner do not appear in both the training and test sets.

### 3.7 Organ classification

Based on the CT‐ORG dataset, we performed a multi‐class organ classification (excluding the brain due to class imbalance) using a MLP, consistent with the classifier used in previous experiments. Performance was evaluated using 10‐fold CV. Although the dataset lacks dose annotations, this setting allowed us to test feature robustness across a range of real‐world acquisition conditions and anatomical variability. Additionally, UMAP was used to project the feature embeddings into 2D space, including the brain class for visualization to inspect its distribution relative to other organs.

## 4 RESULTS

This section presents the results of our analysis, covering feature stability, dose and tissue classification performance, and the structure of the learned latent space. We first report the ICC and CV accuracy to assess feature robustness and sensitivity to dose variations. Then, we visualize the learned representations using UMAP to explore how different factors—including liver tissue class, manufacturer, and dose level—affect the feature distribution. These visualizations help evaluate the separation of anatomical structures and reveal potential biases introduced by acquisition parameters.

We computed ICC per feature with dose levels as fixed raters and 1608 observations (see Section 3.3, Equation (1)). Table 2 summarizes the feature stability and dose classification performance for the four feature extraction methods. Given the near‐balanced number of observations across dose levels (see Table S1), accuracy serves as an appropriate metric for evaluating dose classification performance.

**TABLE 2:** Feature stability and dose classification performance of different feature extraction methods. The ICC represents feature stability across dose levels, computed with dose levels as fixed raters and using all 13 scanners, reconstruction algorithms, ROIs and all 10 repetitions per dose, while CV accuracy indicates dose classification performance. Poor performance in dose classification suggests that dose‐related information is not, or is only weakly, represented in the features. We do not interpret dose classification performance as inherently positive or negative, so no directional arrows are shown in the table.

		Feature stability	Dose classification
Features	# Features	Mean ICC	CV Accuracy
PyRadiomics	86	0.8355 ± 0.1705	0.5234 ± 0.0356
Shallow CNN	2048	0.8416 ± 0.2018	0.5869 ± 0.0397
SwinUNETR	768	0.9528 ± 0.0272	0.3796 ± 0.0250
CT‐FM	512	0.9347 ± 0.0420	0.6517 ± 0.0179

**FIGURE 3:**
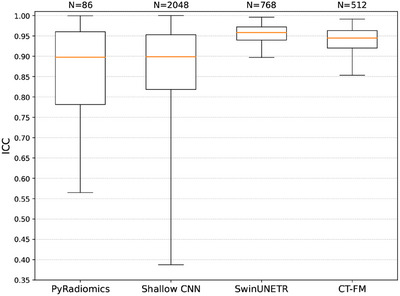
ICC comparison across feature extraction methods with dose levels as raters: PyRadiomics, Shallow CNN, SwinUNETR, and CT‐FM. The number of samples N corresponds to the feature dimensionality of each method.

Figure 3 shows the distribution of ICC values for each feature extraction method, providing insights into feature stability across scanners and reconstruction methods. Additional distinct analyses for various reconstruction methods are provided in Figure S1.

**FIGURE 4:**
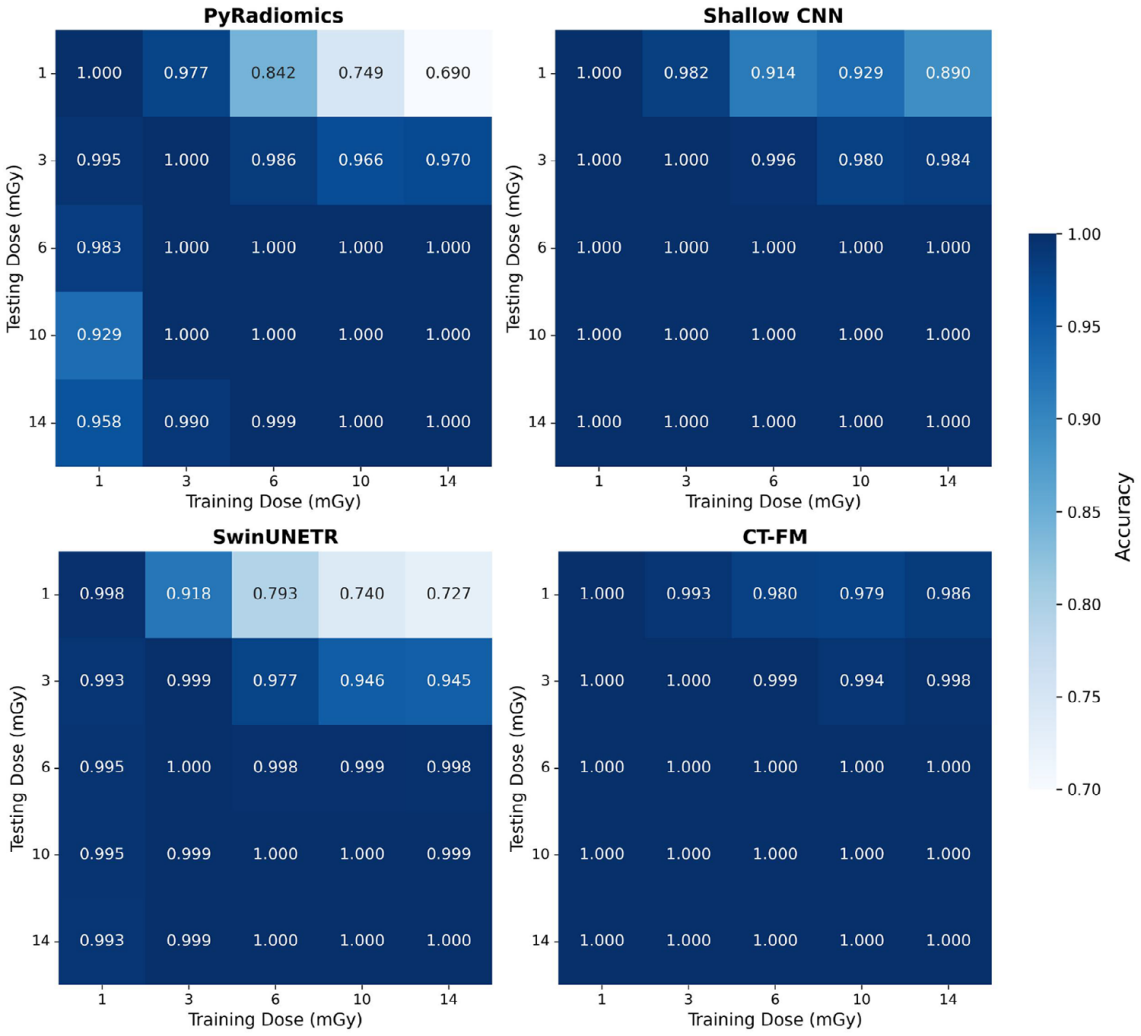
Accuracy matrix for liver tissue classification across various training and test doses.

The results of the liver tissue classification across dose levels are presented in Figure 4, where the matrices show the test accuracy obtained when training on one dose and testing on another. This analysis provides valuable insights into the consistency and robustness of liver tissue classification under varying dose conditions.

**FIGURE 5:**
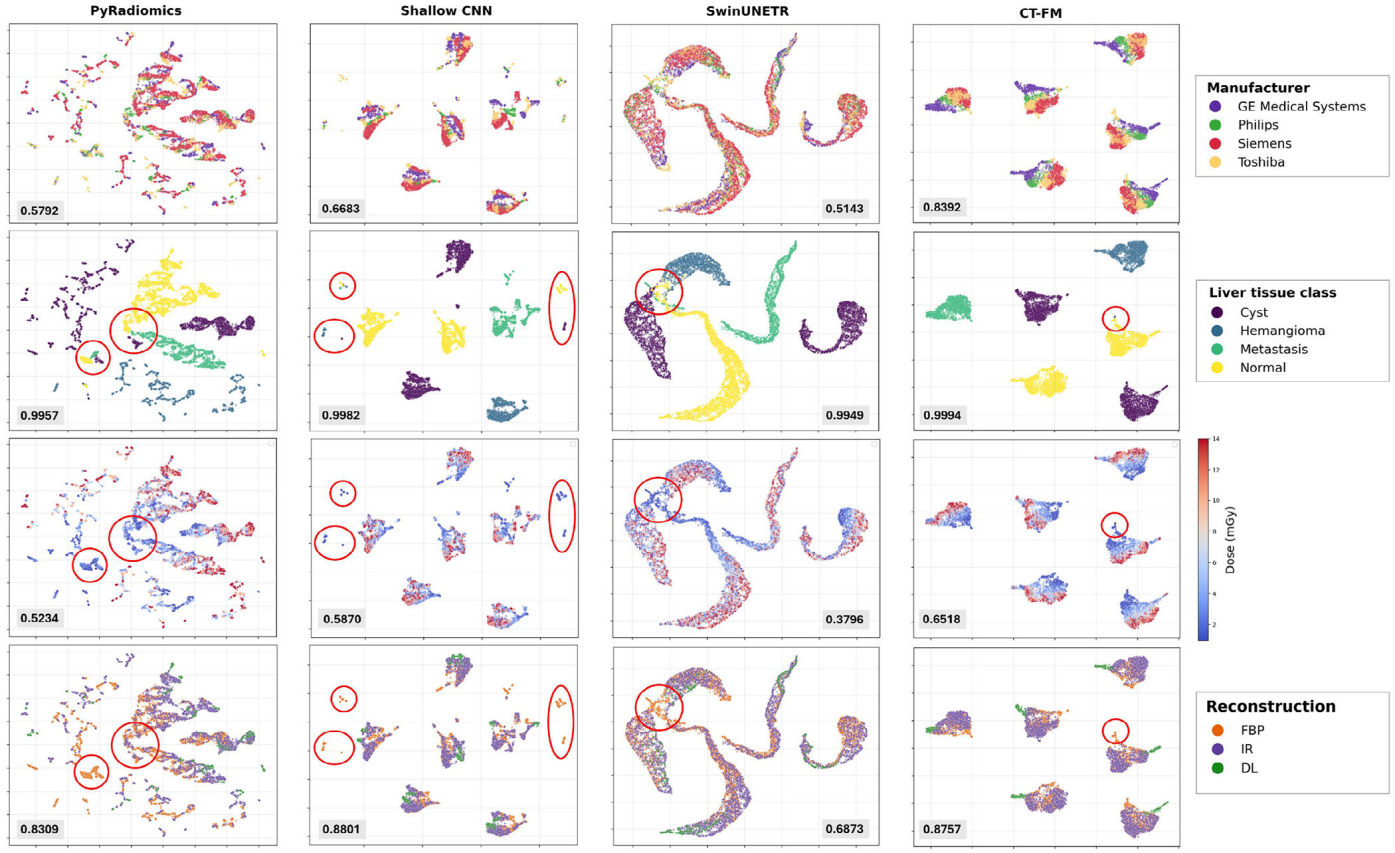
UMAP visualization for the four different feature extraction methods (columns). Each row shows the same UMAP projection colored by a source of variation: (1) scanner manufacturer, (2) liver tissue class, (3) dose, and (4) reconstruction algorithm. The circles highlight method‐specific patterns where lower doses lead to tissue class confusion: in PyRadiomics and SwinUNETR, they mark regions where clusters from different tissues overlap (which correspond to low‐dose areas in the dose‐colored row); in the CNN and CT‐FM projections, they indicate dispersed points farther from their tissue clusters, also aligning with low‐dose regions. For each plot, the corresponding mean classification accuracy is shown; additional details and 95% bootstrap confidence intervals are provided in Table S2.

To analyze the computed representations further, we employed UMAP to explore the structure of the latent space. Figure 5 presents three UMAP visualizations, each highlighting a specific source of variation: scanner manufacturer, liver tissue class, dose level and reconstruction method. These visualizations allow assessing how well the extracted features separate liver tissue classes while also revealing potential biases introduced by the considered sources of variation. Classification performances are reported for liver tissue classification as well as for each source of variation. Table S2 also reports corresponding confidence intervals estimated with bootstrapping.

**FIGURE 6:**
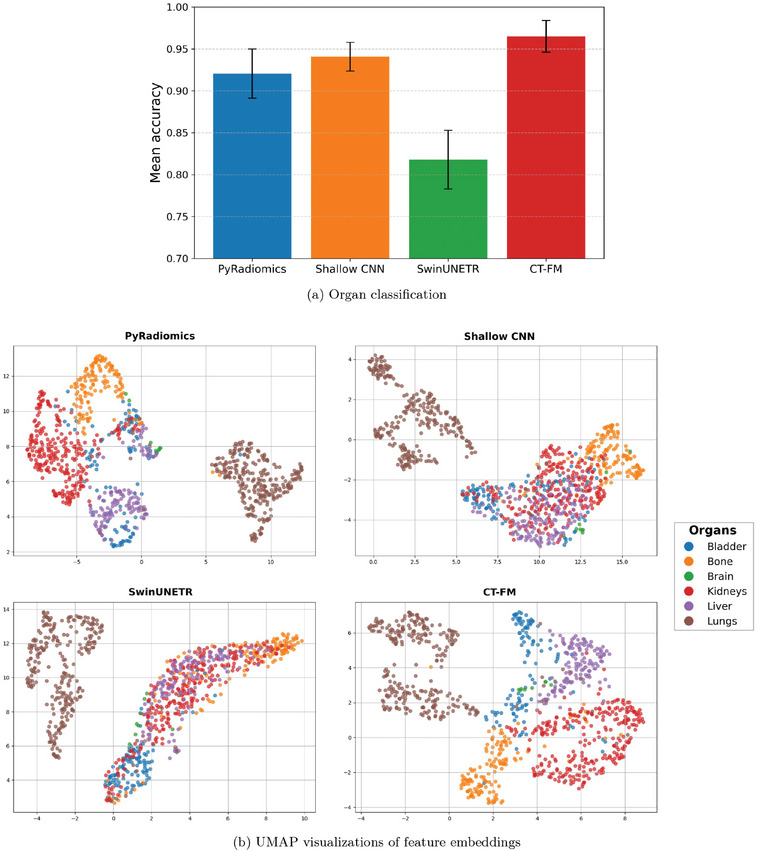
(a) Mean organ classification accuracy and standard deviation from 10‐fold CV on the CT‐ORG dataset using the MLP classifier, comparing different feature extraction methods. (b) UMAP visualizations of feature embeddings from the CT‐ORG dataset.

To estimate feature discriminability in a clinical setting with real patient data, we analyzed the CT‐ORG dataset. The results, shown in Figure 6, highlight the differences in classification performance across feature extraction methods and reveal how well the extracted features capture organ‐specific patterns.

## 5 DISCUSSION

This study investigated how dose variation in CT imaging influences the performance and robustness of AI models, which plays an important role in ensuring reliable predictions in critical applications in precision medicine such as diagnosis, treatment planning, and prognosis. By employing UMAP, ICC and liver tissue classification analyses with an anthropomorphic phantom and a controlled acquisition protocol, we assessed the effectiveness of various methods in capturing dose‐related variability as well as characteristics of liver tissue types.

The ICC analysis revealed that SwinUNETR and CT‐FM exhibited the highest feature stability across dose levels (SwinUNETR: Mean ICC = 0.9528 ± 0.0272, CT‐FM: 0.9347 ± 0.0420), followed by the shallow CNN (0.8416 ± 0.2018), and PyRadiomics (0.8355 ± 0.1705). These findings indicate that DL‐based approaches, particularly models pretrained on larger and more diverse datasets like CT‐FM, extract more consistent and dose‐invariant features than handcrafted radiomic features. Impact of reconstruction algorithm in Figure S1 revealed that FBP reconstruction is the least stable to dose variations.

In the liver tissue classification task (Figure 4), PyRadiomics and SwinUNETR exhibited noticeable accuracy drops across different dose combinations, especially when training on high‐dose features (14 mGy) and testing on low‐dose features (1 mGy). This highlights the challenge of transferring knowledge from high‐dose to low‐dose images, as high‐dose scans offer more detailed features and better contrast, which models may overly rely on, while low‐dose images introduce noise and lower contrast. In comparison, the CNN and CT‐FM consistently achieved near‐perfect accuracy across all dose combinations, which is notable with such extreme dose settings. CT‐FM, in particular, demonstrated superior resilience with its ability to learn generalized, high‐level features that persist across dose levels, likely related to its pretraining on a diverse dataset with varying dose conditions. The impact of classifier type was studied in Table S3, revealing that MLP is consistently the best or second‐best performer across feature sets, and the relative ranking of the four extractors is unchanged. In practice, MLPs are also best representatives of common downstream heads in clinical AI pipelines.

UMAP clustering, shown in Figure 5, reinforced these observations. PyRadiomics and SwinUNETR both showed limited ability to maintain clear tissue class separability in low‐dose images. PyRadiomics displayed the poorest separability overall, with substantial cluster overlap, particularly in low‐dose regions—likely due to its reliance on handcrafted features that struggle with complex spatial patterns and noise. SwinUNETR, on the other hand, achieved generally good tissue clustering, but clusters for different liver tissues in low‐dose images tended to overlap. This overlap decreased at higher doses, where clusters became more distinct. This limited separability in low‐dose regions reflects SwinUNETR's reduced ability to differentiate tissue types, as observed in the liver tissue classification task (Figure 4), where accuracy drops were observed across different dose combinations. When combining all doses together, all methods showed nearly perfect tissue classification performance, highlighting the inherent simplicity of the task limited by the phantom nature of the study.

In contrast, the Shallow CNN and CT‐FM maintained better tissue clustering. While the CNN showed some dispersion in low‐dose areas, highlighted by circles in the UMAP plots, it still largely preserved class distinctions without major overlap. This suggests the model can differentiate between tissue classes, though with less robustness in low‐dose regions. CT‐FM, on the other hand, stood out with the most robust clustering, preserving clear liver tissue separability while revealing a smooth, consistent gradient across dose levels in the dose‐colored UMAP plots. This demonstrates its ability to encode dose‐specific information without compromising tissue separability, suggesting robustness under the dose variation commonly seen in real‐world settings.

These trends were further supported by the dose classification results in Table 2. Overall, the dose classification accuracies are relatively low (≤0.66) across all methods. This is expected since lower accuracy reflects higher invariance of the features across dose levels—that is, features remain consistent despite changes in radiation dose. However, we do not assume that higher or lower invariance is inherently good or bad.

Notably, the clearer separation of dose levels observed in the UMAP embeddings of CT‐FM and the Shallow CNN is consistent with their higher classification accuracies (CT‐FM: 0.6517 ± 0.0179, Shallow CNN: 0.5869 ± 0.0397). In contrast, PyRadiomics and SwinUNETR struggled to separate dose levels in the embedding space, reflected in their lower classification performance (PyRadiomics: 0.5234 ± 0.0356, SwinUNETR: 0.3796 ± 0.0250), indicating a more limited ability to capture dose‐related variation. Overall, we observed a superior capacity of the CT‐FM to implicitly model variations in image acquisition, which is supported by higher classification accuracies of the latter in Figure 5 and Table S2.

Interestingly, CT‐FM achieved the highest dose classification accuracy despite also having high ICC values, which challenges the common assumption that a high ICC implies insensitivity to dose variation. Instead, this suggests that ICC reflects consistency in extracting intra‐tissue features across doses, without preventing the model from encoding dose‐relevant information. In fact, CT‐FM appears to learn a rich feature representation that captures tissue identity, dose level, and even manufacturer—likely along different dimensions of the embedding space. The low intra‐tissue variability (as reflected in tight UMAP clustering) coexists with clear inter‐dose separability, particularly within each tissue type. Conversely, SwinUNETR achieved the highest ICC yet the lowest dose‐classification accuracy, indicating that high ICC can reflect within‐tissue consistency across doses but does not, by itself, determine dose discriminability. These findings underscore the importance of evaluating models with complementary methods—such as dose classification and UMAP visualization—rather than relying solely on ICC to understand model robustness under varying acquisition conditions. Taken together, these observations suggest that relying on ICC alone may provide an incomplete picture of the robustness of foundation models, as it captures only part of the behavior relevant to downstream performance. While feature invariance to for example, dose changes is often considered a desirable property for the robustness of clinically relevant tasks such as tissue classification, our experiments with CT‐FM suggest that it is not strictly required for good performance in this particular model. In our setting, high CT‐FM performance in liver tissue and dose classification co‐exists with dose‐sensitive feature dimensions, with CT‐FM retaining dose information alongside tissue information rather than discarding it, which can be useful for interpretability analyses and dose‐aware applications. It is also worth noting that measuring stand‐alone feature stability with metrics such as ICC does not account for subsequent internal weighting of the features in downstream classifiers, and may therefore assign disproportionate importance to noisy feature dimensions that would be down‐weighted or effectively discarded by adequately trained models.

Although phantom studies enable the control of sources of variation, they have significant design limitations, such as the representation of only one fixed structure, which greatly restricts design options for tasks that are directly clinically relevant. To this end, we extended tissue classification performance analysis to real patient data with the CT‐ORG dataset. It confirmed important trends where the CT‐FM model achieved the highest organ classification accuracy (0.965) and produced the most distinct clustering of organ types in the UMAP embedding space (Figure 6). PyRadiomics and the Shallow CNN followed with slightly lower performance (0.921 and 0.941), while SwinUNETR lagged behind (0.818). The Shallow CNN performs slightly better than SwinUNETR on this task, likely because it was pretrained on a dataset of organs for classification, whereas SwinUNETR was optimized for segmentation and general feature learning. This lower organ‐classification performance of SwinUNETR on CT‐ORG contrasts with its higher ROI‐level classification performance on the phantom dataset (Figure 5), reflecting the increased complexity and heterogeneity of real patient data compared with the controlled phantom setting. These results indicate that CT‐FM features handle heterogeneous clinical data well, suggesting good generalizability across imaging conditions in real patient datasets.

A key factor behind CT‐FM's strong performance is its pretraining on a large and diverse dataset of 148,000 CT scans from multiple institutions, which allows it to learn highly generalizable feature representations. This pretraining enables CT‐FM to effectively adapt to variations in scanner types, acquisition protocols, and dose levels. Although the exact dose information in the training set was unavailable, the dataset's diversity in dose levels likely contributes to CT‐FM's ability to maintain both stability and discriminative power across varying conditions. Furthermore, CT‐FM's contrastive self‐supervised learning approach enhances its capacity to extract spatially consistent and anatomically relevant features. Although its architecture is simpler than segmentation‐oriented networks such as SwinUNETR, CT‐FM's large‐scale pretraining may contribute to better behavior under heterogeneous acquisition conditions. More broadly, our results suggest that leveraging large, diverse datasets and modern self‐supervised methods can help develop representations that are more stable across real‐world variability—an encouraging direction for future, task‐specific clinical evaluations.

Regarding the potential impact of resampling on dose robustness, we did not conduct a dedicated analysis isolating this preprocessing step. In our processing pipeline, the reconstructed CT images were resampled to a common voxel grid with 2mm slice thickness and 0.684mm pixel spacing to standardize voxel dimensions. Because this resampling was applied only to a small subset of scanners, any influence on the reported dose‐related trends is expected to be limited. Quantifying this effect explicitly is a useful direction for future work.

In light of this analysis, we emphasize two takeaways. First, stand‐alone feature stability metrics (e.g., ICC) may not be sufficient on their own as indicators of downstream robustness and are best interpreted alongside task performance. Second, for deployment, we recommend prioritizing foundation‐model pipelines trained on broad, heterogeneous data and aligned to the local target population. This aims to learn real‐world variability—dose, vendors, protocols—rather than depending on heavy harmonization. Reporting performance stratified by dose (and other acquisition factors) and incorporating dose information when available can further help characterize and potentially exploit this variability, while keeping the focus on diverse data and strong pretrained representations to carry robustness into clinical use.

## 6 CONCLUSION

In this study, we explored the impact of CT dose variation on AI models' performance using four different feature extraction methods ranging from hand‐crafted features to foundation models. Our analysis was conducted on a large, multicentric phantom dataset acquired across multiple scanners, with real (non‐simulated) dose reductions. This setup enabled a controlled yet realistic evaluation of how acquisition changes affect model robustness and generalizability. Unlike prior work that often relied on synthetic noise injection, this approach isolates the true impact of dose variability under clinical imaging conditions. We assessed feature stability through ICC and visualized feature distributions with UMAP to understand how each method captures or resists dose‐related variation.

Our findings indicate that radiomic features are more sensitive to dose variations, exhibiting lower ICC values and inconsistent clustering. Both the shallow CNN and SwinUNETR improved feature stability compared to radiomics, but still showed some sensitivity to dose variation. CT‐FM, however, demonstrated the highest robustness, delivering the most consistent performance across dose levels, which was confirmed with real patient data on the CT‐ORG dataset. This resilience is likely due to the foundation model's exposure to diverse imaging conditions during large‐scale pretraining, demonstrating its adaptability to real‐world, heterogeneous environments.

This work highlights the importance of considering dose variability in the development and deployment of AI‐based tools in medical imaging. It also underscores the potential of foundation models to improve the generalizability and reliability of image‐based predictions across variable acquisition protocols. Furthermore, the study emphasizes the value of large, diverse datasets in training robust models that can adapt to different imaging conditions. Future work may explore harmonization strategies, such as contrastive learning or domain adaptation, to further mitigate the effects of dose and scanner variability in clinical settings.

## CONFLICTS OF INTEREST STATEMENT

The authors declare no conflicts of interest.

## Supporting information

Supporting Information

## Data Availability

The CT phantom dataset analyzed in this study is publicly available on The Cancer Imaging Archive (TCIA) under the collection name *CT4Harmonization‐Multicentric*.^12^ The code used for this study is available at https://github.com/mariamartinasiain/radiomics_phantom_dose_analysis.git.
