# Supplementary material for: Impact of CT dose on AI performance: A comparison of radiomics, deep, and foundation models in a multicentric anthropomorphic phantom study
Source: Med Phys. 2026 Mar 18;53(3):e70374. doi: 10.1002/mp.70374 (PMC12997016; doi:10.1002/mp.70374)
Supplement: Supplementary file 1 — Supporting Information [file MP-53-0-s001.pdf]

Appendix A

Table S-1: Number of CT image series acquired from each manufacturer, categorized by acquisition dose and reconstruction algorithms.

		Dose					Reconstruction Algorithm			Overall
**ID**	**Manufacturer and Model**	**1 mGy**	**3 mGy**	**6 mGy**	**10 mGy**	**14 mGy**	**FBP**[a]	**IR**[b]	**DL**[c]	**Series**
A1	Siemens SOMATOM Definition Edge	20	20	20	20	20	50	50	-	100
A2	Siemens SOMATOM Definition Flash	20	20	20	20	20	50	50	-	100
B1	Siemens SOMATOM X.Cite	20	20	20	20	20	50	50	-	100
B2	Siemens SOMATOM Edge Plus	20	20	20	20	20	50	50	-	100
G1	Siemens SOMATOM Definition Edge	20	20	20	20	20	50	50	-	100
G2	Siemens SOMATOM Definition Flash	20	20	20	20	20	50	50	-	100
C1	Philips Brilliance iCT 256	20	20	20	20	20	50	50	-	100
H2	Philips Brilliance CT 64	20	20	20	20	20	50	50	-	100
D1	GE Revolution Evo	30	30	30	20	20	50	50	30	130
E2	GE Revolution Apex	30	30	30	30	30	50	50	50	150
F1	GE BrightSpeed	20	20	20	20	20	50	50	-	100
E1	Toshiba Aquilion Prime SP	20	20	20	18	20	49	49	-	98
H1	Toshiba Aquilion CXL	20	20	20	20	20	50	50	-	100
**Sum**		**280**	**280**	**280**	**268**	**270**	**649**	**649**	**80**	**1378**

[a] Filtered backprojection,
[b] Iterative reconstruction,
[c] Deep learning based reconstruction.

Table S-2: Mean accuracy with 95% bootstrap confidence intervals (MLP classifier) across tasks reflecting different sources of variation: dose, tissue, manufacturer, and reconstruction.

**Task**	**PyRadiomics**	**Shallow CNN**	**SwinUNETR**	**CT-FM**
Dose classification	0.5234 [0.5002, 0.5442]	0.5870 [0.5636, 0.6131]	0.3796 [0.3646, 0.3953]	0.6518 [0.6413, 0.6635]
Liver tissue classification	0.9957 [0.9906, 1.0000]	0.9982 [0.9954, 1.0000]	0.9949 [0.9896, 0.9987]	0.9994 [0.9986, 1.0000]
Manufacturer classification	0.5792 [0.5767, 0.5818]	0.6683 [0.6144, 0.7223]	0.5143 [0.5092, 0.5195]	0.8392 [0.8331, 0.8452]
Reconstruction classification	0.8309 [0.8228, 0.8404]	0.8801 [0.8691, 0.8909]	0.6873 [0.6784, 0.6964]	0.8757 [0.8666, 0.8839]

Table S-3: Comparison of downstream classifiers (mean accuracy ± std, 10-fold CV) for dose classification across feature extraction methods.

**Classifier**	**PyRadiomics**	**Shallow CNN**	**SwinUNETR**	**CT-FM**
LR	0.5197 ± 0.0315	0.4795 ± 0.0332	0.4037 ± 0.0236	0.5846 ± 0.0273
KNN	0.4978 ± 0.0651	0.3293 ± 0.0393	0.3476 ± 0.0244	0.5068 ± 0.0252
RF	0.5293 ± 0.0714	0.4393 ± 0.0393	0.3710 ± 0.0182	0.5739 ± 0.0301
SVM	0.5044 ± 0.0321	0.4944 ± 0.0474	0.3546 ± 0.0284	0.6149 ± 0.0301
MLP	0.5234 ± 0.0356	0.5869 ± 0.0397	0.3796 ± 0.0250	0.6517 ± 0.0179

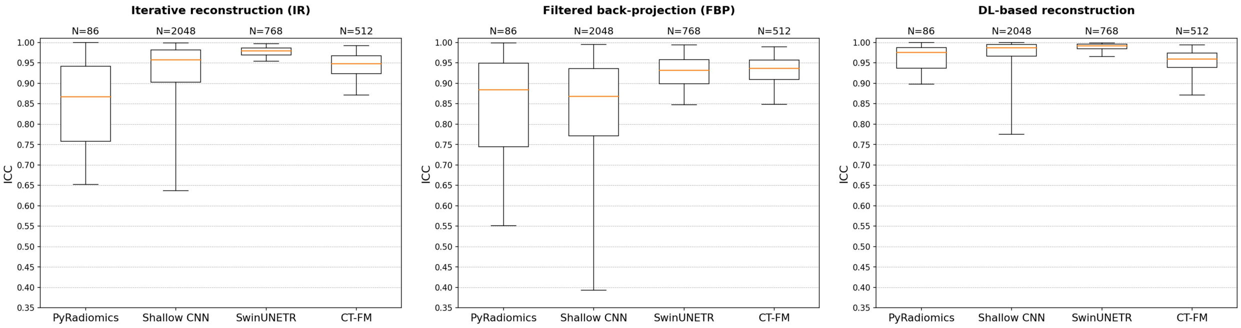

Figure S-1: ICC comparison across feature extraction methods with dose as raters (Eq. 1) for each reconstruction method: IR, FBP and DL-based reconstruction. The number of samples N corresponds to the feature dimensionality of each method.
